# Designing an Intervention to Improve Medication Safety for Nursing Home Residents Based on Experiential Knowledge Related to Patient Safety Culture at the Nursing Home Front Line: Cocreative Process Study

**DOI:** 10.2196/54977

**Published:** 2024-10-09

**Authors:** Marie Haase Juhl, Ann Lykkegaard Soerensen, Henrik Vardinghus-Nielsen, Lea Sinding Mortensen, Jette Kolding Kristensen, Anne Estrup Olesen

**Affiliations:** 1 Department of Clinical Medicine University of Aalborg Aalborg Denmark; 2 Department of Clinical Pharmacology Aalborg University Hospital Aalborg Denmark; 3 University College of Northern Denmark Aalborg Denmark; 4 Department of Health Science and Technology Aalborg University Aalborg Denmark; 5 Senior and Care Municipality of Aalborg Aalborg Denmark; 6 Research Unit for General Practice in Aalborg Aalborg University Aalborg Denmark

**Keywords:** intervention development, nursing home, frontline professionals, medication safety, quality improvement, patient safety culture, experiential knowledge, cocreation, resilient health care systems, safety II perspective, human resources, integrated knowledge translation

## Abstract

**Background:**

Despite years of attention, avoiding medication-related harm remains a global challenge. Nursing homes provide essential health care for frail older individuals, who often experience multiple chronic diseases and polypharmacy, increasing their risk of medication errors. Evidence of effective interventions to improve medication safety in these settings is inconclusive. Focusing on patient safety culture is a potential key to intervention development as it forms the foundation for overall patient safety and is associated with medication errors.

**Objective:**

This study aims to develop an intervention to improve medication safety for nursing home residents through a cocreative process guided by integrated knowledge translation and experience-based codesign.

**Methods:**

This study used a cocreative process guided by integrated knowledge translation and experience-based co-design principles. Evidence on patient safety culture was used as an inspirational source for exploration of medication safety. Data collection involved semistructured focus groups to generate experiential knowledge (stage 1) to inform intervention design in a multidisciplinary workshop (stage 2). Research validation engaging different types of research expertise and municipal managerial representatives in finalizing the intervention design was essential. Acceptance of the final intervention for evaluation was aimed for through contextualization focused on partnership with a municipal advisory board. An abductive, rapid qualitative analytical approach to data analysis was chosen using elements from *analyzing in the present*, addressing the time-dependent, context-bound aspects of the cocreative process.

**Results:**

Experiential knowledge was represented by three main themes: (1) closed systems and gaps between functions, (2) resource interpretation and untapped potential, and (3) community of medication safety and surveillance. The main themes informed the design of preliminary intervention components in a multidisciplinary workshop. An intervention design process focused on research validation in addition to contextualization resulted in the Safe Medication in Nursing Home Residents (SAME) intervention covering (1) campaign material visualizing key roles and responsibilities regarding medication for nursing home residents and (2) “Medication safety reflexive spaces” focused on social and health care assistants.

**Conclusions:**

The cocreative process successfully resulted in the multifaceted SAME intervention, grounded in lived experiences shared by some of the most important (but often underrepresented in research) stakeholders: frontline health care professionals and representatives of nursing home residents. This study brought attention toward closed systems related to functions in medication management and surveillance, not only informing the SAME intervention design but as opportunities for further exploration in future research. Evaluation of the intervention is an important next step. Overall, this study represents an important contribution to the complex field of medication safety.

**International Registered Report Identifier (IRRID):**

RR2-10.2196/43538

## Introduction

### Background

Despite years of focus, medication safety remains a key challenge of health care systems, with the development of effective interventions taking on a critical role in ensuring overall patient safety [1-3]. In the context of primary care, social disparities have been found in nursing home residents, considered a marginalized population at higher risk of avoidable harm in health care [4-6]. Characterized by advanced age and multiple comorbidities, nursing home residents are often subjected to complex medication regimens and polypharmacy [7-9]. These circumstances, while aimed at improving their overall health, paradoxically expose them to heightened risks of medication errors and unsafe medication practices [1,5,10]. Medication dispensing and administration and communication incidents have been reported as main sources of unsafe care among older adults in primary care [11]. In addition, cognitive and physical impairments prevalent in nursing home residents can lead to high dependency on others for their medication management. Thereby, the potential benefits of nursing home residents’ active engagement in their own medication safety may diminish [12]. In Denmark, nursing homes form part of primary care, which is under municipal responsibility [13]. In Danish nursing homes, a nonlicensed delegation of social and health care assistants and helpers represents >80% of the front line, in contrast to hospital settings, where physicians and nurses are more frequently present at the front line. A growing interest in medication safety in primary care has developed in the past years, with reported initiatives aimed at improvement [14]. Nevertheless, studies concerning interventions aimed at improving medication safety focused on residential aged care facilities, including nursing homes, remain scarce and inconclusive [15]. A more recent example involves a multifaceted intervention focused on pharmacists’ medication review and interprofessional collaboration [16]. To assess the potential effects of the intervention, a randomized controlled trial was conducted, reporting no measurable improvement in health status in nursing homes allocated for intervention compared with controls. Overall, earlier studies investigating potential effects of interventions to improve medication safety in primary care remains inconclusive [14,17].

A recognized need for interventions addressing aspects of medication safety beyond prescribing has been shared [18]. Aligning with other Scandinavian countries, general practitioners are overall responsible for medication management for nursing home residents in Denmark [19]. General practitioners conduct medication prescription, whereas final dosage and administration of medication is performed by a nonlicensed delegation of frontline health care professionals, including social and health care assistants. In addition, social and health care helpers are crucial partners in medication management as they count the number of dispensed pills and assess alignment with prescribed medication from the locally shared electronic medication registration system. Nurses are consulted in complex cases while not often being physically present in Danish nursing homes. Nursing home residents themselves also play an important role in medication safety but are often impaired both physically and cognitively. Thus, frontline health care professionals can be critical observants and communicators on behalf of the nursing home residents and play a critical role in medication safety improvement. Moreover, relatives of nursing home residents may serve as their primary advocates with regard to medication safety [5].

Integrated knowledge translation (IKT) provides a cocreative approach to research focused on equally powered partnerships among researchers, knowledge users, and decision makers. Integration of different knowledge sources and implementation are considered essential to IKT [20,21]. This aligns with experience-based co-design (EBCD) principles emphasizing experiential knowledge from end users as critical in developing interventions to address actual needs and experiences of those targeted by the intervention [22]. While a multicomponent intervention design supports the enhancement of medication safety in residential aged care settings, including nursing homes [15], evidence of effects of reported medication safety programs remain limited [14].

Patient safety culture can be defined as “a reflection of professionals’ shared assumptions, values, beliefs, and practices” [23] and is seen as foundational not only for overall patient safety [3,24] but also in the prevention of medication errors [25]. Earlier research has suggested interventions aimed at continuous improvement of organizational culture in long-term care facilities to enhance patient safety [26]. Thus, patient safety culture is a promising avenue for interventions aimed at improving medication safety. According to an umbrella review investigating existing tools to measure organizational culture in health care organizations, tangible themes related to patient safety culture may be assessed using existing quantitative questionnaires [27]. Importantly, the umbrella review further identified 9 intangible themes requiring disentanglement in future studies through the use of qualitative methods. In doing so, potential new insights could be revealed, setting new directions in intervention development aimed at improving medication safety in nursing home residents. Thus, this study emphasized the development of an intervention to improve medication safety for nursing home residents focused on cocreation guided by IKT and EBCD principles and integration of intangible themes related to patient safety culture [27].

### Objectives

This study aimed to develop an intervention to improve medication safety for nursing home residents through a cocreative process grounded in lived experiences of medication safety. This paper covers a cocreative process with the following specific objectives: (1) to generate experiential knowledge on medication safety grounded in evidence of patient safety culture to inform (2) the design of the Safe Medication in Nursing Home Residents (SAME) intervention.

## Methods

### Overall Study Design

#### Overview of the Cocreative Process

This study introduced an innovative approach to intervention development applying a cocreative process guided by IKT and EBCD principles. The cocreative process covered a multistage, combined developmental study design [28]. Taking an integrative stance, results of an initial explorative stage (stage 1) informed subsequent intervention design (stage 2). No predefined intervention target area was established. An abductive strategy was chosen emphasizing an inductive exploratory approach to intervention development, deductively informed by evidence on patient safety culture. IKT principles concerned the integration of evidence on patient safety culture and lived experience in the generation of experiential knowledge of medication safety. Furthermore, the focus on implementation based on partnership formation with a municipal advisory board aligned with the IKT principles. EBCD activities included semistructured focus groups with frontline health care professionals and representatives of nursing home residents and a multidisciplinary workshop. These activities supported a bottom-up leveled intervention development emphasizing marginalized voices besides from a top-down leveled contextualization focused on local nursing home settings. The combination of IKT and EBCD principles in guiding the cocreative process was chosen with the aim of developing an intervention addressing actual needs and experiences of representatives of nursing home residents, encompassing their relatives in addition to frontline health care professionals. At the same time, this combination acknowledged contextualization of the intervention as equally important to intervention design, supporting the translation of research into practice.

**Figure 1:**
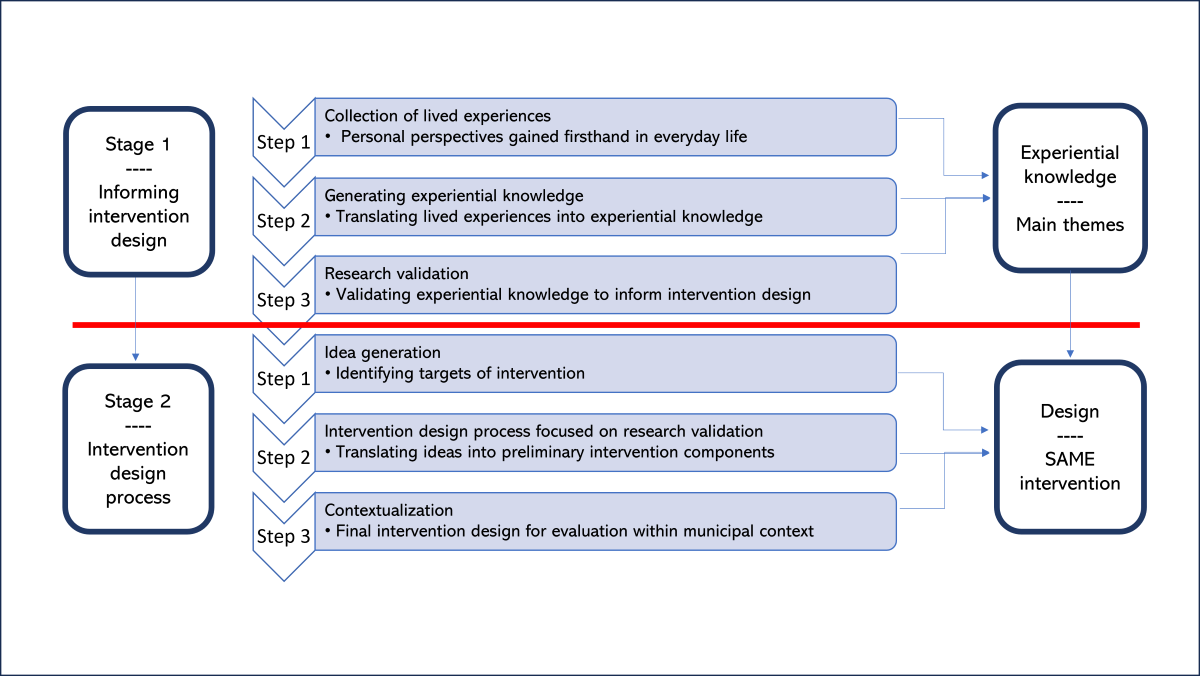
The cocreative process. Illustration of the iterative, integrative, cocreative process of intervention development including the main stages and respective steps. SAME: Safe Medication in Nursing Home Residents.

Regarding work package 1, this study covered a cocreative process embedded within a larger mixed methods research project, the “Safe Medication in Nursing Home Residents” (SAME) study (ClinicalTrials.gov NCT04990986) [29]. The cocreative process is illustrated in Figure 1, with further details provided in this section.

#### Stage 1: Generating Experiential Knowledge on Medication Safety to Inform Intervention Design

Following steps were conducted in stage 1:

- Step 1: conducting exploratory focus groups to collect individual lived experiences from representatives of nursing home residents and nursing home frontline health care professionals
- Step 2: translating these individual experiences into a shared understanding of medication safety for nursing home residents in a triangulated process, forming experiential knowledge represented as main themes
- Step 3: validating the experiential knowledge by researchers from different fields of expertise, including qualitative, quantitative, and participatory research methods to ensure the reliability and validity of the experiential knowledge

#### Stage 2: Designing the Intervention

Following steps were conducted in stage 2:

- Step 1: facilitating idea generation for preliminary intervention components in a multidisciplinary workshop informed by validated experiential knowledge from stage 1
- Step 2: transforming ideas into preliminary intervention components though research validation
- Step 3: contextualization of preliminary intervention components resulting in the final intervention design (SAME intervention)

#### Cocreative Approach

With no definitive standard for cocreation in health care [30], IKT principles were chosen for their historical roots in medicine [21]. IKT aims to facilitate innovation by bridging research and practice through equally powered partnerships, thereby guiding the development of impactful interventions [31]. The integration of different types of knowledge is also considered essential in IKT. In this study, core knowledge included evidence on patient safety culture and experiential knowledge on medication safety . Furthermore, the focus on engaging frontline health care professionals, nursing home residents, and families in intervention development was set as key to intervention development [31,32]. This resonates with the use of EBCD, promoting bottom-up intervention design through participatory research methods such as focus groups and workshops [22]. Therefore, combining IKT and EBCD principles was found to support the development of an evidence-informed intervention that addresses genuine needs while remaining contextually grounded for seamless implementation.

#### Cocreative Activities: Focus Groups and a Multidisciplinary Workshop

Inspired by EBCD, key cocreative activities were covered within the cocreative process, including exploratory, semistructured focus groups to engage nursing home residents, their relatives, and nonlicensed frontline health care professionals. Through semistructured discussions, participants were supposed to share their subjective experiences concerning medication safety, enabling the identification of novel insights and focus areas for intervention.

In addition, a multidisciplinary workshop was used for idea generation drawing on insights from the focus groups. Inspired by the principles of “future workshop” [33], this session aimed to foster critical discussion and creative thinking, building an innovative platform for intervention development. Overall, the focus groups took an organizational perspective on medication safety, emphasizing the importance of the local nursing home environment in realizing interventions, whereas the workshop aimed to achieve a system-level frame of intervention design to support a sustainable intervention with increased generalizability despite local tailoring.

#### Settings

The focus groups and workshop were conducted at the municipal headquarters in Aalborg, Denmark, from April to October 2021. Focus group sessions lasted 3 hours, whereas a 1-day workshop extended over 6 hours, incorporating a lunch break (45 minutes) and smaller breaks (10-15 minutes). All cocreative activities ended with a 15-minute plenum evaluation.

#### Facilitation

The facilitation of the focus groups and workshop was led by an external consultant in cocreativity and communication complemented by researchers from the SAME study [29]. In the focus groups, a researcher participated primarily in generating field notes and audiotapes, whereas 3 researchers served as cofacilitators and field note generators during the workshop. Immediately after the workshop, a shared document capturing researchers’ reflections was produced. The external consultant provided individual written reflections and verbal feedback on researchers’ reflections. The researchers and external consultant played pivotal roles in knowledge translation, integrating research expertise with experiential knowledge related to practical cocreation within municipalities. The external consultant in cocreation and communication had experience as inspirator, coach, and process manager, aiding individuals and organizations in promoting new thoughts, vision, language use, and behavior. The essence of the facilitators’ competencies lay in perspectives of challenges and focus on positive areas of development. The consultant had several years of experience in politics in addition to process management in partnership with Danish municipalities. In addition, a municipal risk manager, also a member of the municipal advisory board, collaborated with the researchers, contributing actively to the focus group evaluation (10 minutes) following the initial assessment.

#### Theoretical Perspectives on Medication Safety

To create a safe environment for sharing lived experiences, prevailing safety I theoretical perspectives and recommended safety II perspectives were presented as part of the initiation of both the focus groups and workshop. A safety I perspective focuses on safety reactively, whereas the safety II perspective is more proactive. A safety II perspective to intervention development highlights humans not only as root causes of errors but also as resources to address continuous change within health care environments. By presenting different perspectives on medication safety, a “safe sharing space” was aimed for to minimize risk of “shame and blame,” thereby supporting more honest and vulnerable sharing of lived experiences and discussion throughout the cocreative process [34].

#### Participating Cocreators

To address the challenge of dependency in nursing home residents, relatives and nonlicensed frontline health care professionals were chosen for inclusion, also taking a pragmatic stance due to time and resource limitations inherent in this study [29]. In total, 4 semistructured focus groups were conducted based on three differing roles: (1) social and health care assistants (n=2); (2) social and health care helpers (n=1); and (3) representatives of nursing home residents, including relatives and not-for-profit organizations concerned with people of older age (n=1). Social and health care assistants’ education focuses on nursing care but also medication administration with a duration of approximately 4 years. Social and health care helpers’ education relates to competences in care and practical service, with emphasis on being able to react properly to changes in patients’ habitual status and communicate with residents and their relatives. Representatives of nursing home residents included members of the Senior Council, Aalborg, Denmark, and the DaneAge Association and relatives engaged in nursing home councils of users and relatives, a particular initiative of the municipality of Aalborg [35]. Nurses of municipal employment, either home care nurses or nursing home nurses, were targeted for inclusion as both groups are implicated in medication for nursing home residents. Unfortunately, the COVID-19 pandemic situation and a general strike held by nurses during the study period did not allow for their recruitment.

#### Overall Recruitment

The recruitment process was grounded in partnership between SAME researchers and the municipal risk manager through the municipal advisory board. Information material was initially developed by SAME researchers, with the municipal advisory board providing subsequent feedback. The municipal risk manager helped in ensuring that the design of the material aligned with municipal design principles before final acceptance was obtained from the municipal advisory board representatives. After acceptance, cocreators were invited via email. Invitations were specifically directed to eligible cocreators but also to their registered nursing home managers to ensure managerial acceptance of the cocreators’ engagement. This included the time expected to be spent on the study to fit with local workloads. In addition, the overarching partnering role of the municipality, with this study being part of the larger SAME study, was specified. Recruitment of representatives of nursing home residents was done through contact with existing groups of relatives present in nursing homes within the municipality of Aalborg, Denmark. The study protocol can be consulted for further details concerning study methods [29]. Table 1 provides an overview of the cocreators and outputs from each stage and step.

**Table 1:** Overview of the Safe Medication in Nursing Home Residents (SAME) intervention development—study stages, steps and related methods, participating and facilitating cocreators, and outputs.

Study stages, steps, and related methods		Cocreators	Outputs
**Stage 1: generating experiential knowledge on medication safety**			
	Step 1: Semistructured focus groups Collecting lived experiences in exploratory focus groups	Nursing home focus group attendees: Social and health care assistants (n=5) Social and health care helpers (n=4) Representatives of nursing home residents and their relatives (n=4) Facilitators: External consultant in cocreativity and communication SAME researcher	Empirical material: Lived experiences on medication safety grounded in patient safety culture
	Step 2: Triangulated analytical process Transforming lived experiences into experiential knowledge in a triangulated process	Internal, multidisciplinary research partners: SAME researchers and external consultant in cocreativity and communication Facilitator: Researcher	Experiential knowledge: Main themes to inform intervention design
	Step 3: Individual feedback sessions Research validation of experiential knowledge to ensure its reliability and validity to inform intervention design integrating external research and clinical field expertise	Individual researchers with expertise in qualitative, quantitative, and participatory research methods and representing multidisciplinary clinical fields, including nursing, medicine, and pharmacology Facilitators: External consultant in cocreativity and communication SAME researcher	Research-validated experiential knowledge: Validated main themes to inform intervention design
**Stage 2: designing the intervention**			
	Step 1—multidisciplinary workshop: Idea generation based on critical reflection including different perspectives Translating ideas into preliminary intervention design	Multidisciplinary workshop attendees: Representatives of nursing home residents (n=2) Social and health care assistants (n=3) Social and health care helpers (n=1) General practitioner dedicated to nursing homes (n=2) Municipal risk manager (n=1) Hospital risk manager (n=1) Assisting municipal leader (nursing home area) (n=1) Representative of nurses (related to nursing homes; n=1) Representative of nursing home manager (n=1) Consultant in general practice (focus on those of older age; n=0)	Idea generation and preliminary intervention components: Ideas of interventions to improve medication safety for nursing home residents and translation of ideas into preliminary intervention components based on whether consensus to support the ideas was obtained from all cocreators participating in the workshop and the SAME researchers
	Step 2—individual intervention design feedback sessions: Intervention design process integrating different fields of research expertise to ensure the reliability and validation of preliminary intervention components integrating external research and clinical field expertise	Internal, multidisciplinary research partners: 3 researchers and 1 external consultant in cocreativity and communication External multidisciplinary research partners with expertise in qualitative, quantitative, and participatory research methods and representing multidisciplinary clinical fields, including nursing, medicine, and pharmacology Facilitators: External consultant in cocreativity and communication Researcher	Validated preliminary intervention components: Validated preliminary intervention components for contextualization
	Step 3—a contextualizing process concerning the municipal advisory board: Focus on equally powered partnership—municipal advisory board Integrating research evidence and management-and leadership-based knowledge; fit intervention to local resource frame(s)	Municipal advisory board partners: Internal research partners: 3 researchers and 1 external consultant in cocreativity and communication Municipal leader and management representatives: Municipal risk manager Assisting nursing home area leaders Nursing home manager Leader of the municipal department of quality and innovation	The SAME intervention: Final intervention design accepted for evaluation within local context

### Overview of the Cocreative Process

#### Stage 1: Generating Experiential Knowledge on Medication Safety to Inform Intervention Design

##### Overview

In stage 1, exploratory, semistructured focus groups were used to generate experiential knowledge represented as main themes to inform subsequent intervention design in stage 2. Main themes structured lived experiences to present a shared understanding of medication safety based on the lived experiences shared in the focus groups, thereby drawing specific attention toward the local context. Addressing this context-bound aspect and real-world focus of this study, “rapid qualitative analysis” (RQA) was found appropriate for data analysis [36]. RQA included principles of listening and relistening to audiotapes as a principal analytical strategy inspired by “Analyzing in the Present” [37].

##### Defining Experiential Knowledge

Experiential knowledge, introducing lived experience as a core type of knowledge in research, played a pivotal role in intervention development, recognizing experiential knowledge’s subjective nature within specific contexts [38]. This type of knowledge, rooted in patient but also professional experiences, contrasts with scientific, factual knowledge commonly referred to as evidence [38]. The emphasis on equally important voices related to experiential knowledge distinguishes it from evidence, which often places end users’ perspectives at a taxonomic bottom [38]. The use of experiential knowledge supported the integration of voices from different subgroups not often represented in evidence centered on scientific knowledge when it comes to targeting interventions to improve medication safety. In this study, perspectives on medication safety shared as firsthand lived experiences by those actively involved in medication safety within the nursing home setting, including representatives of frontline health care professionals and nursing home residents as well as their relatives, were central in informing intervention development.

##### Step 1: Collection of Lived Experience

###### Overview

Initially, lived experiences regarding medication safety were collected in exploratory, semistructured focus groups (step 1). Data included audiotapes and field notes. A semistructured interview guide was developed for data collection in stage 1. As the guide was in Danish, it is not reported in this paper, but essential aspects of its development are presented in the following sections.

###### The Semistructured Interview Guide

A qualitative semistructured interview guide was crafted in Danish structured around 3 levels of exploration encompassing a total of 11 themes. The framework for a qualitative semistructured interview guide was used to design the interview guide [39]. The semistructured interview guide aimed to formulate exploratory, inductive questions with a deliberate emphasis on existing evidence on patient safety culture emerging as a potential key to medication safety and foundational to intervention development in the literature [27]. The literature highlights both tangible (including leadership, teamwork, training and development, patient orientation, employee and job attributes, organizational structures and processes, and communication systems) and intangible (commitment, trust, psychological safety, power dynamics, support, communication openness, blame and shame, moral values, ethical considerations, and cohesion) themes related to tools to measure patient safety culture [27]. With tangible themes related to patient safety culture being readily assessable through existing quantitative measures, intangible themes remained largely unexplored. Recognizing the significance of these uncharted intangible themes, they were prioritized as the primary inspiration for shaping the interview guide, serving as guiding principles rather than definitive frames. This abductive approach allowed for the exploration of novel areas.

An external consultant in cocreation and communication (Table 1) was engaged in the interview guide development. This consultant played a crucial role in refining the wording of the guide and translating evidence into practical language, ensuring clarity and relevance for the intended audience. This collaborative effort aimed to bridge the gap between research evidence and real-world application, enhancing the usability and effectiveness of the interview guide. The final guide underwent pilot-testing within a focus group of social and health care assistants. With no major adjustments required, it focused on 3 main themes—“the challenge,” “the individual,” and “the community”—aligning with the exploratory aim of this study.

##### Step 2: Generation of Experiential Knowledge

###### Overview

In step 2, lived experience was translated into experiential knowledge, represented as main themes (step 2). Experiential knowledge of those actively involved in medication safety in nursing homes refers to a socially produced, shared understanding of medication safety based on subjective, lived experiences.

###### The Semistructured Interview Guide

**Figure 2:**
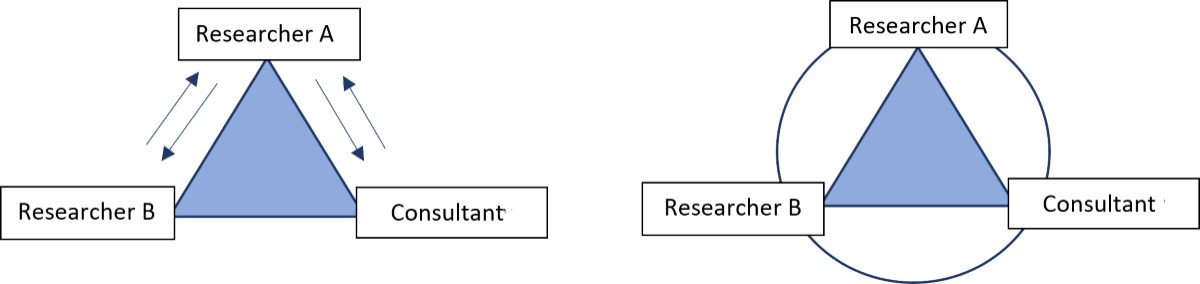
The triangulated analytical process to translate lived experience into experiential knowledge. Initially, researcher A was responsible for gathering individual analyses of all cocreators involved (left). Second, all cocreators met and discussed what was found and collaboratively decided on the outcome (right). External consultant refers to the external consultant in cocreation and communication representing a cocreator in the Safe Medication in Nursing Home Residents intervention.

Translation of lived experiences into experiential knowledge emphasized a triangulated, analytical process in which lived experiences were structured into main themes and related subthemes by researchers and an external consultant in cocreativity and communication, grasping the essence of RQA methodology and the cocreative principles of partnership and multi-source knowledge. The triangulated, analytical process comprised 2 consecutive steps as illustrated in Figure 2.

Therefore, merging lived experience and scientific knowledge in a triangulated analytical process was essential to data analysis. Key elements of the triangulated analytical process are visualized in Figure 2. Thematic analysis was initiated with data collection by the researcher observing the focus group sessions following the RQA approach. After the sessions, the observant researcher (researcher A) and a senior researcher (researcher B) with expertise in qualitative methods, in addition to an external consultant in cocreativity and communication, independently listened to audio recordings from the focus groups. Individual feedback sessions between the researchers and the consultant resulted in the generation of preliminary themes. A subsequent triangulation and shared analysis process occurred. A whole-day workshop was set for SAME researchers and the external consultant using mind-mapping forms, field notes, and triangulated preliminary themes to identify the final main themes representing experiential knowledge accepted for research validation with the purpose of informing intervention design.

####### Step 3: Research Validation

Finally, external researchers from different fields of expertise, including qualitative methods, validated the experiential knowledge. This was done though individual feedback sessions referring to research validation in Figure 1 (step 3). A whole-day session was conducted, with the SAME researcher as facilitator and the external consultant in communication and cocreation as cofacilitator. The research validation process included individual feedback sessions with 3 senior researchers with expertise in different clinically relevant fields (Table 1). Both internal and external multidisciplinary partners including both researchers and clinicians were invited to engage in research validation. Thus, both researchers partnering in the SAME study (cocreators) and researchers not engaged in the cocreative process were invited for inclusion.

###### Stage 2: Designing the Intervention

####### Overview

For intervention design, a multidisciplinary workshop was designed in the second stage divided into 3 iterative steps (Figure 1), described in the following sections. Aspects of “future workshop” design [40] inspired the workshop design, embracing a democratic problem-solving strategy with the purpose of combining different interests, experiences, and positions in generating ideas on future solutions to an existing problem. The workshop was planned to include focus on preparation, introducing the safety II perspective, critique through presentation of experiential knowledge, creative thinking, and implementation through practical implications emphasizing different viewpoints and ideas.

####### Step 1: Idea Generation and Preliminary Intervention Components

The presentation of experiential knowledge was conducted through textual formats. Table 1 provides an overview of cocreators involved in the workshop. Initially, attendees were introduced to the concepts of safety II perspectives and the main themes and insights identified in stage 1. Attendees were organized into smaller, multidisciplinary groups with rotating cofacilitators. Within these groups, participants proposed and discussed ideas related to intervention components. These group discussions were instrumental in generating diverse perspectives and innovative concepts. Subsequently, the proposed intervention component ideas were shared and discussed within the larger workshop setting. A consensus-building evaluation occurred in the plenum, where preliminary intervention components were discussed. Components deemed nonessential based on participant arguments were excluded from further research validation.

####### Step 2: Research Validation

Research validation encompassed internal, external, and local perspectives; knowledge bases; and clinical skills. Internal research validation involved 2 researchers and an external consultant independently analyzing recorded material through a listening and relistening process of the focus group interviews and workshop. Analytical triangulation, as illustrated in Figure 2, involved individual analyses by researcher A and subsequent collaborative decision-making by all cocreators. New, mutually agreed–upon categories emerged during collaborative analysis, further developed in a 1-day session attended by researchers, clinicians and external consultants, leading to the identification of main themes.

External research validation focused on researchers with expertise in different methodologies who did not participate in the workshop being individually presented with step 1 outputs for feedback. In addition, a feedback session was held with participation from an external researcher serving as the head of municipality-related research in addition to a representative (assisting leader of nursing homes) from the municipal advisory board. This feedback session took place to bridge the validation process from internal validation to contextualization.

####### Step 3: Contextualization

The final design of the SAME intervention for further evaluation was done in collaboration with the municipal advisory board. As part of the iterative study design, the municipal research director (Aalborg) attended in a 2-hour face-to-face session with SAME researchers with the aim of giving feedback on the preliminary intervention design, particularly identifying issues related to future implementation and overall municipal values and actual safety improvement strategies. Subsequently, the contextualization involved SAME researchers and the municipal advisory board. The municipal risk manager played a crucial role in information exchange throughout the contextualization process, with meetings involving all partners at multiple points. Emphasis on resource allocation within narrow limitations throughout the contextualization process aimed to strengthen future implementation potential.

#### Ethical Considerations

The North Denmark Region Committee on Health Research Ethics reviewed and deemed the SAME study exempt according to the study design and the emphasis on the sole use of survey, interview, and national register methodology (2020-000992). Participation was voluntary, and informed consent was obtained from all participants and could be withdrawn at any point in time. Privacy and confidentiality protection was successfully ensured. No quotations are presented in this paper to ensure anonymity, with the revised paper including summarized results. Only general practitioners were compensated for participation (950 DKK [US $138.69] per hour) according to the Danish “Agreement on General Practice.” All other participants received no compensation. The study was registered at and approved by the institutional data protection department, Department of Research Data and Statistics, Aalborg University Hospital (2021-015), and in ClinicalTrials.gov (NCT04990986). The study was conducted according to the Declaration of Helsinki (64th World Medical Association General Assembly, Fortaleza, Brazil, October 2013).

## Results

### Key Results

Key results include 3 main themes, representing a shared understanding of medication safety for nursing home residents grounded in the lived experiences of social and health care assistants, social and health care helpers (frontline health care professionals), and representatives of nursing home residents including their relatives. Thus, the main themes centered on the local nursing home environment, presented in this section. Furthermore, key results from stage 2, including ideas and preliminary intervention components, are also presented here. An overview of main themes (stage 1), ideas, and preliminary intervention components (stage 2 [steps 1 and 2]) is presented in Table 2. Due to limited space and to ensure anonymity, the main themes are presented in summarized form, whereas examples of data analysis are available upon request. As an unexpected finding, representatives of nursing home residents were found to reflect “relatives representing nursing home residents,” underscoring peer support rather than the “voice of nursing home residents.” All participants representing nursing home residents were found to have the experience of being a relative. Thus, “relatives representing nursing home residents” was set as the focus in the generation of the results presented in this section.

**Table 2:** Presentation of key results of the cocreative process preceding the final Safe Medication in Nursing Home Residents intervention design, including main themes representing validated experiential knowledge (step 3 in stage 1), ideas (with obtained consensus; step 1 in stage 2), and validated preliminary intervention components (step 2 in stage 2).

Main theme	Ideas	Validated preliminary intervention components
Closed systems and gaps between functions	Social and health care helpers participating in annual health controls with the general practitioner, including medication review Contact person regarding medication specifically Creation of specific, shared “language” extending from the nursing home setting for specific situations related to medication (emergency, daily, or ambulatory) Active engagement of relatives in medication management Feedback to frontline health care professionals ensured regarding adverse event reporting related to medication Guided communication and awareness of the difference between communication and information—visualization of key functions in medication management Professional relatives (“school for relatives”)	Campaign material to visualize key functions in medication management, including general practitioners, nurses, social and health care assistants, and social and health care helpers
Resource interpretation and untapped potential	Awareness of relatives’ expectations regarding engagement in medication management Technological assistants addressing challenges regarding IT solutions iPad in each resident home with ID log-in Inclusion of pharmacist in daily work in nursing homes Physicians specialized in geriatrics as collaborators in medication management Transparency regarding the intervention already offered by the municipality	Self-plot regarding relatives’ expected engagement in nursing home residents’ medication
Community in medication safety and surveillance	Support a positive culture regarding surveillance as collaboration, supporting shared learning across nursing homes Cross-sectoral analysis of errors Less monitoring and more supervision and collaboration—positive aspects of surveillance, including reflexivity in frontline health care professionals	Medication safety reflexive spaces with focus on reflexivity in frontline health care professionals in a reflexive process based on experiences shared by representatives across nursing homes

### Results From Exploratory Focus Groups Experiential Knowledge on Medication Safety

#### Overview

As a result of the exploratory focus groups, experiential knowledge on medication safety to inform intervention design was generated, represented by three main themes: (1) closed systems and gaps between functions, (2) resource interpretation and untapped potential, and (3) community in medication safety and surveillance. In summary, the focus groups revealed key themes regarding medication safety in nursing homes: a closed system of frontline health care professionals versus an open system of relatives, the implicit community in medication safety extending from the nursing home setting, lack of guidance and structured communication, and the potential for untapped resources through enhanced transparency and engagement emphasizing focus on frontline health care professionals and relatives. In the following sections, the 3 main themes are presented.

#### Closed Systems and Gaps Between Functions

This theme highlighted interdependency between specified functions in medication management supporting the creation of a closed system including frontline health care professionals (social and health care assistants and social and health care helpers). Unexpectedly, all representatives of nursing home residents expressed their representation as “relatives” of nursing home residents. The role as “peer support” was reflected in shared experiences rather than representative “voices of nursing home residents.” Thus, “representatives of nursing home residents” shifted to “relatives representing nursing home residents.”

Medication management was identified as a shared challenge among frontline health care professionals, reflecting common values centered on patient safety and the goal of avoiding harm. Social and health care assistants and helpers consistently associated “patient safety” and “medication safety” with avoiding harm, exemplified by one assistant who emphasized the importance of not causing harm to patients. In contrast, relatives had varied perspectives, with some prioritizing happiness over strict involvement in medication processes and others expressing a need for clear guidelines on how to effectively participate in medication management. Frontline health care professionals viewed their roles as interdependent and clearly defined, creating a stark contrast with the relatives’ less defined roles. This discrepancy resulted in a gap between the 2 groups, indicating a closed system among nursing home professionals and a separate, open system among relatives in terms of safety culture. A critical aspect of this closed system was the use of silence in communication to avoid conflicts, particularly when relatives posed questions or demands about medication that professionals felt unprepared to address. This strategy of purposeful silence, driven by a perceived risk of conflict, was explicitly mentioned by health care professionals. Relatives felt excluded from communication, aligning with the professionals’ use of silence. For instance, professionals shared experiences of feeling threatened by relatives’ questions, leading to avoidance of engagement to prevent conflict. Better guidelines and support to foster effective communication was experienced as a clear need in improving medication safety across all focus groups.

#### Resource Interpretation and Untapped Potential

This theme focused on the untapped potential within the nursing home environment, emphasizing the need to translate available resources into practical assets to improve medication safety. Participants recognized the importance of maximizing the use of existing resources to address the limited resources within health care. Untapped potential was identified as a key area for improvement, with innovative interpretation and use of existing assets seen as promising avenues for enhancing medication safety. Participants defined resources broadly, encompassing anything useful for addressing gaps in medication safety. They unanimously called for a key person to bridge knowledge and practice in medication management, highlighting the need for systematization and transparency. Untapped potential was identified in the lack of clear roles and responsibilities for social and health care helpers and relatives, which increased communication risks. Enhancing transparency and addressing unspoken concerns were seen as crucial steps to bridge gaps between functions, fostering a cohesive medication safety system and minimizing conflicts and safety hazards. The role of the nursing home general practitioner was seen as important in bridging information and knowledge gaps, although their physical absence in daily activities was noted. In contrast, relatives’ active engagement was experienced to be important as both relatives and frontline health care professionals had experience of being physically present within the nursing home setting. Frontline professionals valued physical space and time for peer discussions to reduce perceived risks of condemnation and mistrust, indicating that the physical presence of relatives was perceived as a threat in communication for health care professionals, aligning with the finding of silence in communication by frontline health care professionals when relatives sought active engagement. Thus, engagement of relatives was warranted when other collaborators were not available, with emphasis on communication between nursing homes and other health care settings. However, active engagement of relatives in general was not warranted by frontline health care professionals due to a perceived risk of conflict. For example, one assistant mentioned the lack of a private meeting space, making it difficult to discuss sensitive issues without involving residents.

#### Community in Medication Safety and Surveillance

This theme emphasized the importance of a community in medication safety, which was experienced as essential. Focus on general practitioners, nurses, social and health care assistants, and social and health care helpers in addition to relatives was identified. Focus group participants generally perceived a robust collaboration within the nursing home setting among general practitioners, social and health care helpers, and social and health care assistants. This collaboration fostered a sense of community and psychological safety, contributing positively to medication safety. However, nurses and relatives were not perceived as key partners in medication management by frontline health care professionals. Frontline health care professionals noted that the perceived lack of key partnership with nurses and relatives might be related to the physical absence of nurses during routine care as they were often only present during emergent situations. Consequently, communication with nurses was not readily addressed, aligning with their general nonpresence within the nursing homes. In contrast, general practitioners dedicated to nursing homes, who were employed a couple of years before, significantly improved the collaborative experience. Relatives, on the other hand, were included in the shared experience of the community, aligning with their physical presence in the nursing home. They considered nurses as crucial partners in information sharing, which supported trust between relatives and the nursing home organization. Relatives valued the essential information nurses provided but noted that it was often limited to emergent cases, identifying this as a safety issue. The psychological safety experienced by frontline health care professionals contrasted with some relatives’ experiences of distrust toward these professionals, indicating a mismatch in experiences. In addition, the focus group representing relatives revealed heterogeneous experiences and did not reach a consensus, aligning with the first main theme of varied perspectives and engagement in medication safety.

The collaboration among general practitioners, social and health care helpers, and social and health care assistants formed a “community of medication safety.” However, communication issues were notable, particularly with nurses, and there was a lack of guidelines for interprofessional communication. The differing “languages” used by various professionals in decision-making highlighted the power dynamics in verbal communication as those who could articulate their arguments most effectively often influenced the course of action. This further underscored the need for better defined collaboration structures and support to foster effective communication between health care professionals and relatives.

### Results From the Multidisciplinary Workshop

#### Idea Generation and Preliminary Intervention Components

We enlisted a total of 14 cocreators to actively engage in the workshop. An overview of the cocreators is presented in Table 1. Several ideas were shared in the workshop, including a key frontline health care professional engaged specifically in medication for nursing home residents, active engagement in medication management by relatives, technological employees to assist in using local technological solutions (without resource frame), a council of relatives (already in place), integration of pharmacists into daily work with medication (not found useful by general practitioners, who had overall responsibility for medication management), representing a few that were not leveraged for research validation of the reasons listed. Clearly, focus on 3 key ideas was supported: “Visualizing key roles and responsibilities in medication management,” “Visualize the self-reported expectation/need of relatives of nursing home residents to be involved/engaged in the medication management process,” and “Medication safety reflexive spaces to support frontline healthcare professionals to share experiences related to medication safety with peers across nursing homes.” Thus, the agreed-upon ideas focused on increased transparency to support communication openness regarding medication safety and relatives as an untapped potential while acknowledging heterogeneity in relatives’ own perceived function as relatives; and reflexivity in frontline health care professionals to support collaborative, continuous learning across nursing homes as keys to medication safety.

#### Step 2: Intervention Design Process Focused on Research Validation

##### Overview

Resulting from the workshop, the specific ideas with obtained consensus were the following three preliminary intervention components: (1) “Visualization of key roles and responsibilities in medication management,” (2) “Self-plot regarding relatives’ own expected engagement in nursing home residents’ medication,” and (3) “Medication safety reflexive spaces,” with elementary descriptions provided in this section. These preliminary intervention components represented untapped potential to be translated into resources through intervention implementation, focused on transparency in medication management, guided communication emphasizing relatives, and supported collaborative learning and sharing of experiences related to medication safety across nursing homes, in addition to frontline health care professionals and relatives identified as key untapped resources.

##### Visualization of Key Roles and Responsibilities in Medication Management

To visualize key roles and responsibilities in medication management, campaign material, including posters, flyers, and badges outlining key functions, was identified as a preliminary intervention component. Focus on opening communication regarding questions to be asked and potential answers to be expected aimed to minimize interruptions in daily work related to medication management in addition to minimizing misunderstandings based on lack of transparency and knowledge regarding medication for nursing home residents. Fostering trust and psychological safety through guided communication regarding medication management was hypothesized to lead to improvement in medication safety. The campaign material focused on a relatively generic design to enable use across a range of health care organizations, including nursing homes, geriatric departments, general practices, and other settings implicated in medication for nursing home residents. This preliminary intervention component covered an idea of the campaign material, realized through contextualization in step 3.

##### Self-Plot Regarding Relatives’ Own Expected Engagement in Nursing Home Residents’ Medication Management

A tool for relatives to reflect on and visualize their expectations of engagement in medication management to guide health care professionals in communication was identified as a preliminary intervention component. Acknowledging the heterogeneity in experiential knowledge regarding medication safety found in stage 1 led to a visualization of “expectations of engagement,” an initially important step to integrate relatives as active partners in medication safety in future improvement efforts. The concept of the self-plot for relatives aimed to facilitate clarity regarding expected engagement in medication management among relatives, supporting communication between them and health care professionals. A simple plot on paper provided as part of existing introductory interviews taking place in relation to initiation of nursing home residency was chosen to address potential language difficulties present in frontline health care professionals. In this way, minimization of wording was aimed for reflecting the experience of “monitoring” and “registration” representing timely aspects of medication safety by participants in the workshop. This component addressed the heterogeneous values and perceptions among relatives regarding their own involvement in medication management and education across disciplines. This heterogeneity led to direct engagement of relatives found not to be possible as an intervention focus. Furthermore, 3 different areas of the plot were defined to include emergency, ambulatory, and daily medication.

##### Medication Safety Reflexive Spaces

The preliminary intervention component of “medication safety reflexive spaces” was validated through research and accepted for further contextualization. Detailed design elements were refined and incorporated into the final SAME intervention design. These spaces were intended to transform surveillance from a negative element to a positive learning capacity, enhancing the interdependency and communication between social and health care assistants and helpers. The concept of “Medication safety reflexive spaces” emerged as a preliminary intervention component addressing all 3 main systems identified in the study. These reflexive spaces are presented in Multimedia Appendix 1 and relate to main theme 3, “Community in Medication Safety and Surveillance.” The idea of “Learning Reflexive Spaces,” focused on collaborative learning across nursing homes, was suggested in relation to this theme. However, this preliminary intervention component also addressed the other main themes, integrating a comprehensive approach to medication safety.

Addressing closed systems and gaps between functions, medication safety reflexive spaces aimed to bridge the closed systems and gaps between functions through awareness of their existence within the nursing home setting. The focus was on frontline health care professionals, particularly social and health care assistants, and social and health care helpers, who were identified as an untapped resource and represented a closed system. This intervention component emphasized the need for these professionals to be recognized and used effectively within the organizational framework of nursing homes. Another critical aspect of the medication safety reflexive spaces was the emphasis on positive values in surveillance. The intervention component was designed to promote collaborative learning across different nursing homes by focusing on positive feedback mechanisms among frontline health care professionals. This approach was rooted in the idea that positive reinforcement can significantly enhance medication safety practices. The design of the medication safety reflexive spaces aimed to included activities to support open communication and learning among frontline health care professionals. Workshop results highlighted the importance of focusing on these professionals and fostering a culture of “positive surveillance.” This approach encouraged sharing of individual lived experiences to inform collaborative learning efforts. Furthermore, a question aimed at promoting reflexivity through positive feedback was presented: “Can you please provide me an example of something that I have done well regarding medication management today.” This question, inspired by a safety II theoretical perspective, aimed to shift the focus from identifying failures to recognizing and building on daily successes following workshop discussion related to main theme 3. This shift in perspective was intended to generate new experiences that could further inform reflexivity among participants in the medication safety reflexive spaces.

#### Step 3: Contextualization of Validated Preliminary Intervention Components

##### The SAME Intervention

Following research validation, contextualization resulted in the final SAME intervention design, detailed in the following sections. Overall, the cocreative process led to the development of a multifaceted, nontechnological intervention. This intervention comprises both a structural component (campaign material visualizing key roles and responsibilities in medication management) and a reflexive component (medication safety reflexive spaces). Combined, these components were hypothesized to increase transparency regarding actual functions in medication management and promote reflexivity among frontline health care professionals. This approach aimed to enhance communication openness and collaborative learning, supporting trust and psychological safety across nursing homes, thereby improving medication safety for nursing home residents through patient safety culture.

The intervention components were designed to work in tandem but reflect individual aspects to promote medication safety. Supporting a shift from a sole focus on failures to recognizing and learning from successful processes in medication management was emphasized. This approach included the incorporation of positive feedback as a crucial element. The interplay between these components was suggested to improve medication safety for nursing home residents by fostering a patient safety culture grounded in open communication and psychological safety. The partnership between knowledge users (frontline health care professionals and relatives) and stakeholders (municipal managerial representatives and researchers) in decision-making was a key aspect of the intervention’s design. Notably, focus on social and health care assistants and the municipal risk manager as active participants in the multidisciplinary workshop and also partnering in the contextualization process aligned with the cocreative IKT and EBCD principles.

##### The Structural Component Visualizing Key Roles and Responsibilities in Medication Management

###### Overview

The core of the structural component “transparency” is campaign material to support potential questions related to medication. Furthermore, it visualizes the key roles of health care professionals and their responsibilities related to medication management with a focus on setting the right direction of questions potentially generated by all persons implicated in nursing home residents’ medication.

The material was developed for use as both posters and folders. To further increase transparency, badges were also designed, representing the four key roles—(1) general practitioner dedicated to nursing homes, (2) nurses, (3) social and health care assistants, and (4) social and health care helpers—also presented in the other campaign material. The material was developed to enable implementation within local nursing home settings yet holding generic aspects, supporting its implementation within nursing home settings but also for use in other health care settings implicated in the medication of nursing home residents. These, including geriatric hospital departments, general practices, and municipal home care.

###### Planned Diffusion

The campaign material was aimed to be physically presented as wall posters across nursing homes, with a focus on common areas within individual nursing homes. Folders were to be shared and explained when initiating residency at a scheduled introduction meeting in which new residents but also relatives are invited to take part. Badges were to be worn by the represented health care professionals daily.

##### The Reflexive Component

###### Medication Safety Reflexive Spaces

**Figure 3:**
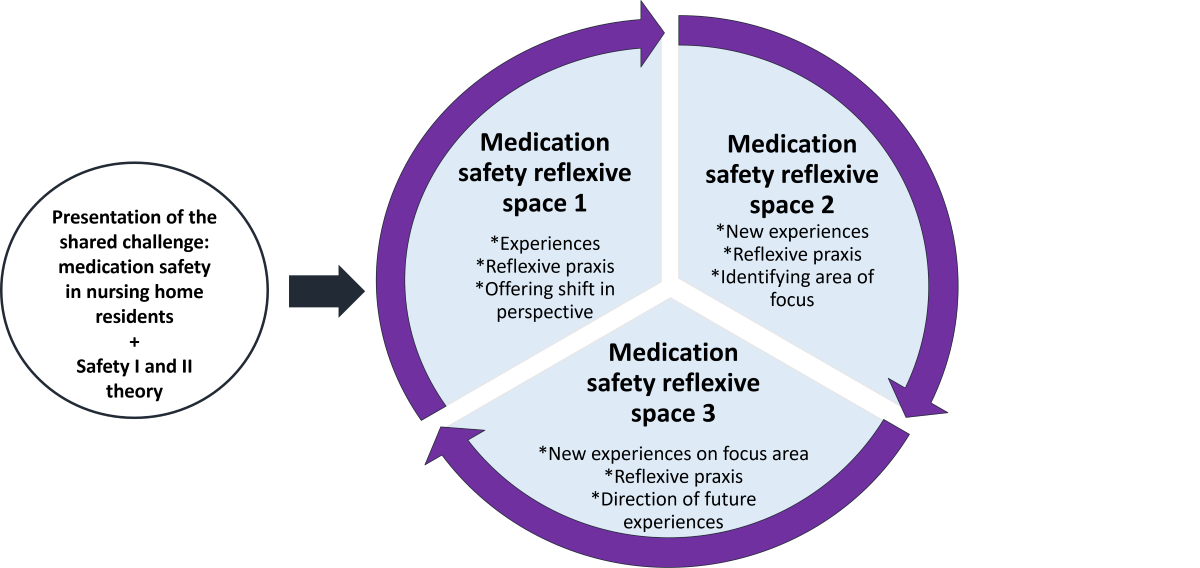
Overview of the generic model of the reflexive component (component 2) of the Safe Medication in Nursing Home Residents intervention consisting of 3 iterative medication safety reflexive spaces. The improvement of medication safety in nursing home residents was hypothesized through continuous, collaborative learning supporting reflexivity in social and health care assistants based on diverse perspectives, including theoretical perspectives on medication safety (safety 1 and 2 theory). The focus on experiential knowledge was set as a key in medication safety improvement. The asterisks indicate concepts being “Shared”.

The reflexive component of the SAME intervention covers a reflexive process grounded in theory on experiential learning, presented in Figure 3. Integrating 3 medication safety reflexive spaces facilitated within neutral, external settings, supporting action within individual nursing homes in between sessions, the reflexive component aimed to support reflexivity in social and health care assistants, hypothesizing that reflexivity can realize medication safety improvement as a mediator of patient safety cultural change. As social and health care assistants represented managing partners to social and health care helpers involved in medication dosage, focus on this group was emphasized by both researchers and the municipal advisory board, representing nursing home leaders. Thus, the reflexive component aimed to increase reflexivity in nonlicensed health care professionals while also addressing a closed system within the nursing home front line, as indicated in stage 1.

The overall intervention covered 3 medication safety reflexive spaces, all integrating the experiences of those participating but holding different aspects. Figure 3 illustrates the overall design of the reflexive component as an overall iterative intervention process, integrating a safety II perspective focusing on sharing experiences and shared decision-making on a focus area to generate new experiences. This process is hypothesized to support reflexivity based on awareness of one’s own and others’ assumptions and critical reflection within a group of professionals holding shared roles and responsibilities across different nursing home units. “Lived experience” was essential to initiate the reflexive process.

As reflexivity includes critical reflection, a key element of the reflexive component is to visualize different perspectives, introducing both actual safety I and recommended safety II perspectives to medication safety. Moreover, experiential knowledge shared by those actively involved in medication safety at the nursing home front line is hypothesized to be key to medication safety improvement. Therefore, to initiate the reflexive intervention process, findings from the focus groups were presented. This was done to induce sharing of diverse, vulnerable lived experiences, aligning with facilitation of the workshop covered in this paper.

###### Transforming Facilitation

Facilitation of the “medication safety reflexive spaces” by an experienced researcher and municipal risk manager is core to the reflexive intervention component. As part of the third reflexive space, the risk manager was set as a cofacilitator to increase future feasibility and implementation in addition to participation in shared evaluation at the end of each session. Each session calls for action by participants, encouraging their experiences to be critically reflected upon both within the “medication safety reflexive space” and as part of ongoing clinical practice. This is to support transformation from awareness of one’s own assumptions and behavior toward reflexivity through shared, critical reflection based on individual lived experience. Importantly, each session’s output depends on the input delivered, whether it be existing or new experiences of participants (Figure 3).

###### Initiation of Medication Safety Reflexive Spaces Supporting a Safe Space

Before sharing experiences, the shared challenge of medication safety improvement for nursing home residents was presented, including introducing safety I theory as the usual perspective to address the problem. Safety II perspectives were then presented to offer an alternative perspective supporting a focus on successes experienced to be shared.

###### Theory of Change

The theory of change of the reflexive intervention component focuses on behavior change related to challenging current practices through a safety II perspective and focus on an area experienced as being of importance to frontline health care professionals actively involved in medication safety in nursing homes. With reflexivity being a contextually bound concept, the iterative process with continuous participation in both “safe spaces” outside the nursing home context and active practice supported in daily clinical practice is essential to the reflexive intervention component.

Thus, “medication safety reflexive spaces” involves integration of external facilitation and local contextualization, supporting reflexive praxis. Focus on frontline health care professionals includes social and health care assistants in focus as participants. The term “reflexive space” is conceptualized as a physical platform for social and health care assistants to engage in critical reflection based on their own lived experiences across different nursing homes. The iterative conduct of the individual intervention sessions, allowing for active experimentation within local nursing home environments, is key to translate reflection into reflexivity. To keep attention toward medication safety, presentation of experiential knowledge (main themes) from stage 1 covered in this paper was chosen to initiate medication safety reflexive spaces. In Figure 3, an overview of the 3 iterative “medication safety reflexive spaces” is presented. Further descriptions of the individual sessions are provided in Multimedia Appendix 1.

###### Setting and Time Schedule

A relatively neutral location situated within municipal headquarters was chosen for conduct of the medication safety reflexive spaces. These headquarters are situated within beautiful nature surroundings, within easy reach and including free parking. All reflexive spaces were held during work hours (daytime) based on partnering with the municipal advisory board. In total, 3-month period and a duration of 3 hours of each intervention sessions, including a light meal and smaller breaks, was decided upon. A total of 15 minutes at the end of each session was assigned to evaluate the shared experiences and participation. This including a municipal risk manager to support further implementation of the intervention.

###### Eligible Participants

Eligible participants were social and health care assistants permanently employed in a public nursing home by the municipality of Aalborg and with experience in the professional field and medication management of >3 months.

## Discussion

### Principal Findings

The cocreative process resulted in the development of a multifaceted, nontechnological intervention with both structural and reflexive components. This supports the need to look beyond technological aspects of health care innovation and underscores the importance of human resources within nursing home settings to support safe medication practices. The multifaceted, contextualized intervention includes the visualization of key roles and responsibilities in medication management and the establishment of “medication safety reflexive spaces” aiming to improve medication safety for nursing home residents.

### Comparison With Prior Work

In the literature, a clear gap in knowledge of interventions to improve medication safety in nursing homes exists. Regarding interventions to enhance safety culture among nursing home professionals in long-term care, studies are also limited, but researchers have found the inclusion of collegial exchange of experiences and learnings, integration of staff’s perceptions, external facilitation, staff training, and a structured multistep procedure of the intervention process to be promising approaches [41]. These aspects were all integrated into this study. a recently published study using a cocreative approach resulted in the creation of reflexive spaces in hospitals with collective sharing of experiences among hospitals, next of kin, and health care professionals to support collaborative learning and cocreation of resilient health care supporting the cocreation of the reflexive component of the developed intervention [42]. Taken together, the results indicate that cocreation could lead to new but aligned directions across health care sectors, with a need for reflexivity to be integrated into safety improvement work, as stated by cocreators in both this study and in hospitals [42]. Therefore, as we cannot conclude how such reflexive practice should be put in place to succeed and lead to positive effects on medication safety, evaluation of the intervention is highly warranted.

### Medication Management Process and Transparency

The structural component of the intervention is designed to enhance clarity regarding roles and responsibilities. With the purpose of improving communication, this intervention component could facilitate more effective and precise interactions, thereby possibly reducing failures in the medication management process. Moreover, this component supports the articulation of questions and directs them to the appropriate professional subgroups, minimizing potential interruptions, a previously discussed risk hazard in medication safety [43]. In addition, unclear knowledge concerning specific roles and responsibilities potentially drives the development of unrealistic expectations relatives, as found in this study, addressed by the structural intervention component of the SAME intervention. Transparency is acknowledged to support safety improvement in healthcare [44]. This element promotes transparency by visually depicting the roles and responsibilities of multidisciplinary health care professionals who, while collaborating, may lack daily direct interaction in nursing home settings. An essential outcome of increased transparency is the promotion of awareness of the fact that questions can and should be posed. Furthermore, offering patients more realistic expectations of care has been suggested as potentially beneficial for reducing threats to patient safety in primary care [39].

### Addressing Key Challenges in Intervention Development

#### Overview

Developing interventions in health care poses various challenges, necessitating the integration of voices representing nursing home residents, including relatives in addition to frontline health care professionals—an imperative acknowledged in the existing literature [45]. In addition, concerns were acknowledged regarding the potential disconnection between research and the practical needs of health care service users. To address possible hierarchical power imbalances within health care organizations [46] and promote diversity, a bottom-up, iterative cocreative process was implemented. This aligns with recommendations from a 2021 narrative review on interventions to optimize medication use in nursing homes. The review suggested conducting large-scale evaluations of underresearched intervention components and interventions addressing medication use aspects beyond prescribing, among other recommendations [18].

#### Using a Combined Cocreative Approach: Focus on Patient Safety Culture, Marginalized Voices, and Implementation

In the pursuit of enhancing medication safety for nursing home residents, this study adopted a cocreative approach guided by IKT and EBCD principles [20,22]. This required collaborative efforts between knowledge users and researchers (stakeholders), emphasizing partnership building spanning the entire cocreative process. The collaboration encompassed the identification of key priorities, the formulation of research inquiries to be responsive to real-world needs, the interpretation of findings, and the facilitation of the practical application of research outcomes [16]. It is noteworthy that IKT differs from conventional knowledge translation methods in its emphasis on cocreation, shared decision-making, and the integration of different types of knowledge and evidence [22,23]. The IKT principles were chosen due to their origin in medicine and recognized significance in supporting research aimed at addressing health disparities and improving health care service delivery. It is important to acknowledge that the existing empirical evidence substantiating the impact of these principles remains limited [20,47].

#### External Facilitator Integration

An external facilitator experienced in communication and innovation within municipal settings in Denmark played a vital role in the cocreative process. This individual was not merely a facilitator but an integral cocreative partner who actively contributed to the design, facilitation, and knowledge generation processes. Nevertheless, the partnership between the external consultant and the research team did also generate potential for disruption. This related to unlearning [48-50] as an innovative key strategy used by the external consultant partnering with SAME. As there is no gold standard of collaborative learning within health care organizations, engaging other types of stakeholders could have led to other innovative strategies, but more knowledge within this field is needed. This study underscores the importance of future focus on external consultants being integral partners in cocreation, reflecting actual practice in improvement work within primary care settings. External consultants may or may not have research expertise, challenging the research aspect of this study. Nevertheless, the research validation and feedback sessions integrating researchers with a wide range of expertise within quantitative, qualitative, and cocreative research fields addresses this issue. In fact, the integration of the external consultant in cocreation and communication in this study may be essential to the open and honest sharing experienced as part of the cocreative process, playing a supportive, neutral role between research and clinical practice. The external facilitator could play a key role in actively using IKT principles to develop equally powered partnerships. Nevertheless, this study remains inconclusive regarding this matter of facilitation, which is why clearly so much more research is needed.

#### Future Perspectives

##### Focus on Collaborative Learning: Medication Safety Reflexive Spaces

While technological interventions are often highlighted to address resource constraints in health care, this study resulted in a nontechnological intervention, emphasizing the significance of knowledge and communication factors. The development of safe reflexive spaces as component 2 of the intervention warrants further evaluation to understand its implications in meeting the need for a safe space expressed by cocreators. The integrative approach, acknowledging diverse perspectives, was crucial. Recognizing variations among organizational subgroups and tailoring interventions to their experiences enhances contextual relevance. The generic design of the medication safety reflexive spaces could allow for their use in various nursing homes, but also in other health care settings, expanding the intervention’s reach. A qualitative study on safe administration of medication in a sample of Norwegian nursing homes found that physical distance between rooms dedicated to medication management represented a possible barrier to safe medication administration, though related interruptions and double checking adaptive behavior [51]. In this study, a general lack of physical room dedicated to medication was experienced, yet it was perceived to be needed by frontline health care professionals. As the resources of this study did not allow for extended physical rooms in nursing homes, allocating a room for medication safety could be a facilitator and not a barrier despite the physical distance being even greater than that in the Norwegian study. In addition, diffusion across different professions or involving residents and their relatives could amplify the impact of medication safety reflexive spaces, potentially influencing health care at a system level.

##### The Importance of Relatives

Cocreation supports the integration of perspectives across a wide range of people, including patients (nursing home residents in this study). Earlier researchers have called for integrating the voices of patients and their relatives in assessing organizational cultural aspects in health care [27]. Supporting nursing home residents’ communication of their needs through the integration of representative voices informing intervention development in this study is a strength, supporting research focused on the improvement of patient safety in primary care [39]. Nevertheless, the “voices of nursing home residents” resulted in the identification of “relatives representing nursing home residents.” Furthermore, the results indicated that relatives represented a separate, open system, with no specific function in medication management. This challenges communication and active engagement of relatives in medication safety efforts, otherwise supported and valued at municipal and national levels concerning patient safety [2]. The open system refers to the results of this study indicating that relatives do not form an integral part of the nursing home front line, challenging the identification of agreement across focus groups, potentially limiting consensus in the workshop. At the same time, this could have increased the innovative potential of the cocreative process, supporting the inclusion of challenging perspectives, reflecting the societal environment external to the nursing home organization. Integrating relatives’ experiences into the workshop could have led to more realistic expectations of areas susceptible to intervention through the identification and presentation of different perspectives, also representing a promising mechanism [39]. Focus on relatives as target of intervention was identified to hold untapped potential in the workshop and resulted in an initial development of a preliminary intervention component. Nevertheless, this intervention component was not prioritized for further contextualization. Although not included for further evaluation in the frame of the SAME study, focus on relatives’ own expected level of engagement in medication management could be an important field of future exploration, with the preliminary intervention component excluded from contextualization representing a starting point.

##### Attention Toward Closed System in Medication Safety

The importance of tailoring interventions to specific subcultures has been proposed as a potential key to improvement [52]. Furthermore, a key to medication safety improvement could lie within the concept of systems, as identified in this study, also referred to as subcultures. Subcultures are present within organizational cultures, including patient safety cultures [53]. If subcultures close upon themselves, it might represent a safety hazard not readily measurable through quantitative instruments. Thus, subcultures, or closed systems, warrant further investigation, including qualitative in-depth inquiry. Furthermore, focus on different cultural languages, including conceptions and silence, could be an important aspect of exploration with potential regarding improvement of medication safety in nursing home residents. This align with earlier quantitative research supporting the existence of subcultural aspects of patient safety culture in nursing homes [54]. Furthermore, a qualitative study in Swedish hospitals suggested that the intervention be tailored to both registered nurses’ and nurse assistants’ patient safety–related assumptions, values, and norms [55]. Altogether, these studies support the final design of the SAME intervention, with initial focus on social and health care assistants, addressing the potential power of a profession-related subculture in need of target before a broader target of intervention, where decision-making could potentially be limited by power hierarchies indicated to be present related to systems found in this study.

##### Supporting the Development of Resilient Health Care Systems

The cocreative approach combined IKT and EBCD principles, representing an innovative approach to intervention development. This study not only adds to a growing body of interventions to enhance medication safety for nursing home residents but also supports knowledge translation into action in the field of resilient health care. Importantly, implementation was integrated into the cocreative process, grounded in the municipal advisory board and contextualization as a final step in intervention design. The SAME intervention is constructed from a structural and a reflexive component targeting aspects other than prescribing, which is one of the most researched aspects in the field of medication safety [15,18]. It cannot be ruled out as a potential consequence of using a cocreative approach. The results support those of recent research emphasizing the important role of collaborative learning in health care focused on reflexive spaces in hospital settings as a resilient health care capacity [42,56]. Furthermore, the reflexive intervention component supports principles for developing learning tools to help translate resilience into practice. Thus, the SAME intervention holds potential for resilience.

### Strengths and Limitations

The strengths of this study lie in its innovative cocreative approach, which fosters critical discussion and creative thinking, resulting in an intervention grounded in lived experiences and patient safety culture. Nevertheless, this innovative approach also constitutes an important limitation with the limited use of classic methods in this study. The inclusion of nursing home residents’ voices, albeit through representatives, provides a realistic depiction of medication safety issues. Engaging relatives has an additional innovative potential, challenging a potential “closed system” of health care professionals representing the nursing home front line. Integration of diverse health care professionals and partnership with the municipal advisory board enhance implementation potential. Addressing sensitivity through a safety II perspective fosters open communication. However, limitations include the exclusion of nursing home residents themselves, potentially hindering the depth of insights. Subjectivity in perceived complexity and single-session focus groups may limit data richness. Generalizability beyond the specific context should be approached cautiously. Nevertheless, generic elements in both the cocreative process and the resulting SAME intervention hold promise for adaptation and future use within other health care settings. Resource constraints and time limitations, along with the absence of nurses as cocreators, pose challenges. In addition, while the study demonstrates cocreation, it falls short as individuals with lived experience as nursing home residents were not part of the research team.

### Conclusions

The cocreative process successfully resulted in the multifaceted SAME intervention grounded in lived experiences shared by some of the most important but often underrepresented stakeholders in research: frontline health care professionals and representatives of nursing home residents. This study brought attention toward closed systems related to functions in medication management and surveillance, not only informing the SAME intervention design but also as opportunities for further exploration in future research. Evaluation of the intervention is an important next step. Overall, this study represents an important contribution to the complex field of medication safety.

### Data Availability

The data sets generated during and analyzed during this study are not publicly available because of the sensitive nature of the information and personal data shared by participants but are available from the corresponding author on reasonable request.

## Appendix

Multimedia Appendix 1 Description of the individual medication safety reflexive spaces.

## Abbreviations

EBCD: experience-based co-design
IKT: integrated knowledge translation
RQA: rapid qualitative analysis
SAME: Safe Medication in Nursing Home Residents

## References

[1] Donaldson LJ, Kelley ET, Dhingra-Kumar N, Kieny MP, Sheikh A (2017). Medication without harm: WHO's third global patient safety challenge. Lancet.

[2] Ageing and health. World Health Organization.

[3] Global patient safety action plan 2021-2030. World Health Organization.

[4] Cheraghi-Sohi S, Panagioti M, Daker-White G, Giles S, Riste L, Kirk S, Ong BN, Poppleton A, Campbell S, Sanders C (2020). Patient safety in marginalised groups: a narrative scoping review. Int J Equity Health.

[5] Bates DW, Zebrowski J (2022). Medication safety in nursing home patients. BMJ Qual Saf.

[6] Piccardi C, Detollenaere J, Vanden Bussche P, Willems S (2018). Social disparities in patient safety in primary care: a systematic review. Int J Equity Health.

[7] Lundby C, Jensen J, Larsen SP, Hoffmann H, Pottegård A, Reilev M (2020). Use of medication among nursing home residents: a Danish drug utilisation study. Age Ageing.

[8] Astorp J, Gjela M, Jensen P, Bak RD, Gazerani P (2020). Patterns and characteristics of polypharmacy among elderly residents in Danish nursing homes. Future Sci OA.

[9] Reilev M, Lundby C, Jensen J, Larsen SP, Hoffmann H, Pottegård A (2019). Morbidity and mortality among older people admitted to nursing home. Age Ageing.

[10] Assiri GA, Shebl NA, Mahmoud MA, Aloudah N, Grant E, Aljadhey H, Sheikh A (2018). What is the epidemiology of medication errors, error-related adverse events and risk factors for errors in adults managed in community care contexts? a systematic review of the international literature. BMJ Open.

[11] Cooper A, Edwards A, Williams H, Evans HP, Avery A, Hibbert P, Makeham M, Sheikh A, J Donaldson L, Carson-Stevens A (2017). Sources of unsafe primary care for older adults: a mixed-methods analysis of patient safety incident reports. Age Ageing.

[12] Manias E, Bucknall T, Hutchinson A, Dow B, Borrott N (2021). Resident and family engagement in medication management in aged care facilities: a systematic review. Expert Opin Drug Saf.

[13] Stuart M, Weinrich M (2001). Home is where the help is: community-based care in Denmark. J Aging Soc Policy.

[14] Khalil H, Shahid M, Roughead L (2017). Medication safety programs in primary care: a scoping review. JBI Database System Rev Implement Rep.

[15] Alruthea S, Bowman P, Tariq A, Hinchcliff R (2022). Interventions to enhance medication safety in residential aged-care settings: an umbrella review. Br J Clin Pharmacol.

[16] Krause O, Wiese B, Doyle IM, Kirsch C, Thürmann P, Wilm S, Sparenberg L, Stolz R, Freytag A, Bleidorn J, Junius-Walker U (2019). Multidisciplinary intervention to improve medication safety in nursing home residents: protocol of a cluster randomised controlled trial (HIOPP-3-iTBX study). BMC Geriatr.

[17] Junius-Walker U, Krause O, Thürmann P, Bernhard S, Fuchs A, Sparenberg L, Wollny A, Stolz R, Haumann H, Freytag A, Kirsch C, Usacheva S, Wilm S, Wiese B (2021). Drug safety for nursing-home residents-findings of a pragmatic, cluster-randomized, controlled intervention trialin 44 nursing homes. Dtsch Arztebl Int.

[18] Spinewine A, Evrard P, Hughes C (2021). Interventions to optimize medication use in nursing homes: a narrative review. Eur Geriatr Med.

[19] Alftberg Å (2022). Medication management in Swedish nursing homes: an ethnographic study of resistance, negotiation and control. Eur J Soc Work.

[20] Gagliardi AR, Berta W, Kothari A, Boyko J, Urquhart R (2016). Integrated knowledge translation (IKT) in health care: a scoping review. Implement Sci.

[21] Nguyen T, Graham ID, Mrklas KJ, Bowen S, Cargo M, Estabrooks CA, Kothari A, Lavis J, Macaulay AC, MacLeod M, Phipps D, Ramsden VR, Renfrew MJ, Salsberg J, Wallerstein N (2020). How does integrated knowledge translation (IKT) compare to other collaborative research approaches to generating and translating knowledge? learning from experts in the field. Health Res Policy Syst.

[22] Green T, Bonner A, Teleni L, Bradford N, Purtell L, Douglas C, Yates P, MacAndrew M, Dao HY, Chan RJ (2020). Use and reporting of experience-based codesign studies in the healthcare setting: a systematic review. BMJ Qual Saf.

[23] Kristensen S, Sabroe S, Bartels P, Mainz J, Christensen KB (2015). Adaption and validation of the safety attitudes questionnaire for the Danish hospital setting. Clin Epidemiol.

[24] de Bienassis K, Kristensen S, Burtscher M, Brownwood I, Klazinga NS Culture as a cure: assessments of patient safety culture in OECD countries. Organization for Economic Co-operation and Development.

[25] Rodziewicz Tl, Houseman B, Vaqar S, Hipskind Je (2024). Medical Error Reduction and Prevention.

[26] Świtalski J, Wnuk K, Tatara T, Miazga W, Wiśniewska E, Banaś T, Partyka O, Karakiewicz-Krawczyk K, Jurczak J, Kaczmarski M, Dykowska G, Czerw A, Cipora E (2022). Interventions to increase patient safety in long-term care facilities-umbrella review. Int J Environ Res Public Health.

[27] Malik RF, Buljac-Samardžić M, Akdemir N, Hilders C, Scheele F (2020). What do we really assess with organisational culture tools in healthcare? an interpretive systematic umbrella review of tools in healthcare. BMJ Open Qual.

[28] O'Cathain A, Croot L, Sworn K, Duncan E, Rousseau N, Turner K, Yardley L, Hoddinott P (2019). Taxonomy of approaches to developing interventions to improve health: a systematic methods overview. Pilot Feasibility Stud.

[29] Juhl MH, Soerensen AL, Kristensen JK, Johnsen SP, Olesen AE (2023). Safe medication in nursing home residents through the development and evaluation of an intervention (SAME): protocol for a fully integrated mixed methods study with a cocreative approach. JMIR Res Protoc.

[30] Grindell C, Coates E, Croot L, O'Cathain A (2022). The use of co-production, co-design and co-creation to mobilise knowledge in the management of health conditions: a systematic review. BMC Health Serv Res.

[31] Gainforth HL, Hoekstra F, McKay R, McBride CB, Sweet SN, Martin Ginis KA, Anderson K, Chernesky J, Clarke T, Forwell S, Maffin J, McPhail LT, Mortenson WB, Scarrow G, Schaefer L, Sibley KM, Athanasopoulos P, Willms R (2021). Integrated knowledge translation guiding principles for conducting and disseminating spinal cord injury research in partnership. Arch Phys Med Rehabil.

[32] Banner D, Bains M, Carroll S, Kandola DK, Rolfe DE, Wong C, Graham ID (2019). Patient and public engagement in integrated knowledge translation research: are we there yet?. Res Involv Engagem.

[33] Carlsson J, Eriksson LO, Öhman K, Nordström EM (2015). Combining scientific and stakeholder knowledge in future scenario development — a forest landscape case study in northern Sweden. Forest Policy and Economics.

[34] Scott-Cawiezell J, Vogelsmeier A, McKenney C, Rantz M, Hicks L, Zellmer D (2006). Moving from a culture of blame to a culture of safety in the nursing home setting. Nurs Forum.

[35] Vedtægter for bruger- og pårørenderåd på plejehjem. Aalborg Municipality.

[36] Taylor B, Henshall C, Kenyon S, Litchfield I, Greenfield S (2018). Can rapid approaches to qualitative analysis deliver timely, valid findings to clinical leaders? a mixed methods study comparing rapid and thematic analysis. BMJ Open.

[37] Revsbæk L, Tanggaard L (2015). Analyzing in the present. Qual Inq.

[38] Halloy A, Simon E, Hejoaka F (2023). Defining patient's experiential knowledge: who, what and how patients know. A narrative critical review. Sociol Health Illn.

[39] Hays R, Daker-White G, Esmail A, Barlow W, Minor B, Brown B, Blakeman T, Sanders C, Bower P (2017). Threats to patient safety in primary care reported by older people with multimorbidity: baseline findings from a longitudinal qualitative study and implications for intervention. BMC Health Serv Res.

[40] Alminde S, Warming H (2019). Future workshops as a means to democratic, inclusive and empowering research with children, young people and others. Qual Res.

[41] Garay S, Haeger M, Kühnlein L, Sulmann D, Suhr R (2023). Interventions to enhance safety culture for nursing professionals in long-term care: a systematic review. Int J Nurs Stud Adv.

[42] Wiig S, Aase K, Bal R (2021). Reflexive spaces: leveraging resilience into healthcare regulation and management. J Patient Saf.

[43] Odberg KR, Hansen BS, Aase K, Wangensteen S (2018). Medication administration and interruptions in nursing homes: a qualitative observational study. J Clin Nurs.

[44] Kolb DA, Cross Jr RL, Israelit SB (2019). The process of experiential learning. Strategic Learning in a Knowledge Economy.

[45] O'Cathain A, Croot L, Duncan E, Rousseau N, Sworn K, Turner KM, Yardley L, Hoddinott P (2019). Guidance on how to develop complex interventions to improve health and healthcare. BMJ Open.

[46] Noyes AL (2021). Navigating the hierarchy: communicating power relationships in collaborative health care groups. Manag Commun Q.

[47] Graham ID, Kothari A, McCutcheon C, Integrated Knowledge Translation Research Network Project Leads (2018). Moving knowledge into action for more effective practice, programmes and policy: protocol for a research programme on integrated knowledge translation. Implement Sci.

[48] Peschl MF (2019). Unlearning towards an uncertain future: on the back end of future-driven unlearning. Learn Organ.

[49] Rushmer R (2004). Unlearning in health care. Qual Saf Health Care.

[50] Esen A, Asik O, Ege T (2019). Unlearning in organizations: understanding and rethinking the way organizations learn and change. PressAcademia.

[51] Odberg KR, Aase K, Olsen RM, Sletvold H (2015). Facilitators and barriers to safe medication administration in nursing homes. Medication Safety in Municipal Health and Care Services.

[52] Tocco Tussardi I, Cazzoletti L, Zanolin ME, Comini A, Visentin D, Torri E, Tardivo S, Moretti F (2023). Patient safety subcultures among nursing home staff in Italy: a cross-sectional study. Healthcare (Basel).

[53] Mannion R, Davies H (2018). Understanding organisational culture for healthcare quality improvement. BMJ.

[54] Deilkås EC, Hofoss D, Hansen EH, Bondevik GT (2019). Variation in staff perceptions of patient safety climate across work sites in Norwegian general practitioner practices and out-of-hour clinics. PLoS One.

[55] Danielsson M, Nilsen P, Ohrn A, Rutberg H, Fock J, Carlfjord S (2014). Patient safety subcultures among registered nurses and nurse assistants in Swedish hospital care: a qualitative study. BMC Nurs.

[56] Lyng HB, Macrae C, Guise V, Haraldseid-Driftland C, Fagerdal B, Schibevaag L, Wiig S (2022). Capacities for resilience in healthcare; a qualitative study across different healthcare contexts. BMC Health Serv Res.

