# Supplementary material for: Designing an Intervention to Improve Medication Safety for Nursing Home Residents Based on Experiential Knowledge Related to Patient Safety Culture at the Nursing Home Front Line: Cocreative Process Study
Source: JMIR Form Res. 2024 Oct 9;8:e54977. doi: 10.2196/54977 (PMC11499722; doi:10.2196/54977)
Supplement: Multimedia Appendix 1 [file formative_v8i1e54977_app1.docx]

#### Safe medication reflexive space 1: Sharing experience, reflexive praxis and offering shift in perspective

To foster reflexive collaborative learning, sharing existing experiences is supported by two main mediating actions with the notion of three keywords or sentences recalled by participants (Poster). Also, a fictive case story of a nursing home resident treated with polypharmacy is presented to induce sharing. To generate new experiences through a Safety II perspective, participants are asked to initiate positive dialogue concerning their work with medication by asking the question: “What have I done well today, regarding medication management” to a collaborator implicated in medication management. This is to initiate dialogue and introduce positive feedback and self-reflection, leading to awareness of other perspectives and potential for improvement.

#### Safe medication reflexive space 2: Sharing of new experiences, reflexive praxis, identify area of focus

To identify areas perceived in need of focus improvement by participants, this session is to be informed by the sharing of new experiences initiated by the question from the first session. Here, the use of reverse role-play is used to guide discussion leading to reflection. To mediate action, three keywords/sentences of medication safety are again exemplified by participants (Poster). Furthermore, shared identification of an area perceived in need of focus for improvement is key to this session. The use of reverse role-play is used to guide discussion leading to reflection. Participants are allocated the role of either general practitioner, nurse, Social- and healthcare assistant, Social- and healthcare helper, nursing home resident- or relative reflecting a case story/experience shared. Roles are meant to shift, to induce increased reflection based on own assumptions of others’ perspectives. Exploring existing knowledge on the identified area by individual participants in each nursing home is then the action called for to inform the last session.

#### Safe medication reflexive space 3: Sharing experiences related to an identified area of focus, reflexive praxis and direction of future experiences

The third session is informed by participants' experiences related to the identified area (Session 2). Keywords or sentences on medication safety are to be presented again with a summary and potential change in these over the three sessions provided by the facilitator. Then, the participants should collectively gather the shared knowledge, if possible, generating a shared product to be disseminated across nursing homes.
